# Predictors of Physical Activity in Middle Childhood. A Fixed-Effects Regression Approach

**DOI:** 10.3389/fpubh.2018.00305

**Published:** 2018-10-24

**Authors:** Tonje Zahl-Thanem, Silje Steinsbekk, Lars Wichstrøm

**Affiliations:** ^1^NTNU Social Research, Trondheim, Norway; ^2^Department of Psychology, Norwegian University of Science and Technology, Trondheim, Norway

**Keywords:** physical activity, moderate and vigorous physical activity, middle childhood, sedentary behavior, community sample, predictors, accelerometer

## Abstract

**Background:** Moderate-to-vigorous physical activity (MVPA) has a range of health benefits across the life span. Although many putative determinants of children's MVPA have been identified, their causal status is uncertain due to difficulties in adjusting for potential confounders.

**Objective:** To inform promotion of children's MVPA we therefore aimed to examine well-known child-, family- and contextual predictors of MVPA in middle childhood, by applying a fixed effects regression approach, which rules out the influence of all unmeasured time-invariant confounders.

**Methods:** Two birth cohorts of children living in the city of Trondheim, Norway were invited to participate (82.0% consented). The participants were followed-up biennially from age 6 to 10 years (*n* = 800) between 2009 and 2014. MVPA in children was recorded by accelerometers and child-, family- and contextual factors were obtained through interviews and questionnaires. Predictors included (i) *child-level factors:* the child's time outdoors, organized sports participation, athletic self-concept, self-reported screen time and objectively measured sedentariness; (ii) *family factors:* self-reported parental MVPA and actively transporting the child to school; and (iii) *contextual factors:* parental socio-economic status (SES), access to playgrounds and ballparks, traffic safety, and having a garden. A three-wave prospective study was conducted with a hybrid fixed effects regressions analysis adjusting for all time-invariant confounders to examine predictors of MVPA.

**Results:** Children evidenced increased MVPA when they spent more time outside, spent less time being sedentary and when the family had a garden and lived in a traffic-safe area.

**Conclusion:** Adjusting for measured time-varying and all unmeasured time-invariant confounders renders many previously identified child and family factors without impact on MVPA in children. However, several contextual factors may promote MVPA in middle childhood, and efforts to facilitate children being outside in environments that promote physical activity (e.g., being outside, in gardens, or otherwise traffic safe areas) may prove important.

## Introduction

Physical activity (PA), and moderate to vigorous physical activity (MVPA) in particular, reduces mortality (1) and protects against a range of disorders and precursors to disorders in adults (2). Such beneficial effects are also observed in children and adolescents (3). Because PA tracks from childhood to adulthood (4), increasing children's PA may increase PA in adulthood and in both developmental epochs promote health, reduce morbidity, and lower the mortality risk in adulthood.

Ideally, such preventive efforts should be guided by research on the factors responsible for promoting or impeding PA in children. Important steps have been taken in this respect, including research on the significance of child, family (5), and contextual (6) factors, such as screen use (7), and time outdoors (8, 9), passive (motorized) transportation (8), and physical aspects of the neighborhood (10).

The variety of potential causal factors examined in the investigations just cited strongly indicates that childhood PA is multi-determined, and that an ecological approach (11) that considers child, family, and contextual factors is called for. However, presumed risk factors within and between contextual levels are often correlated [e.g., traffic safety and access to recreational areas such as ballparks (10)] and parents who are themselves physically active often move to areas that promote and support such activity (12). Consequently, potential causal factors from various arenas should be investigated, and adjusted for each other, a methodological requirement that has not always been met in the above-cited literature. If appropriate adjustments are not made, the risk of mis-specifying the relationship between risks/promoters and MVPA is immanent. However, if rigorous adjustments *are* carried out, even very long lists of covariates cannot eliminate all possible confounding. To illustrate, physical activity is heritable (13). Thus, it is difficult to attribute any causality to any association between parental variables (e.g., parental MVPA, active transporting the child) and offspring MVPA. Such unmeasured 3rd variables will also include common method effects (e.g., parents reporting on both their own and child PA or time spent outdoors), and environmental factors influencing both parental and child PA (e.g., socio-economic disadvantage, environmental attributes and seasonal changes). Of note, the lack of adjustments for real observed and unobserved confounders will inevitably imply that associations will be inflated.

Although a multitude of correlates of children's MVPA have been identified in previous research, the lack of adjustment implies that our knowledge base to build preventive measures on is less certain than perhaps first believed. To overcome some of the problems of unmeasured confounders, we used the fixed effects approach, a methodology that accounts for all unmeasured time-invariant confounders (e.g., genetics, stable parenting characteristics, common method effects) when examining prospective associations (14, 15) which we have successfully applied in several recent papers (16, 17). More specifically, in the present study we estimate the effects of child-, family-, and contextual factors on objectively measured MVPA, net of any more or less time-invariant factors such as genetics, stable parental and family characteristics, and potential reporting bias.

The contexts and types of PA varies with children's age (18), and total PA levels are declining during early school years (19). Understanding predictors of PA in children entering school age and beyond is therefore essential in order to promote PA throughout childhood. Among factors intrinsic to the child, results from previous cross-sectional and some prospective reports show that level of PA is affected by athletic self-concept (20), sedentary screen time (7), time outdoors (21), and participation in organized sports (22). As concerns family factors, studies also suggest that PA is promoted by comparatively higher parental socio-economic status (SES) (21), parental PA (23), parents spending time outdoors with the child (24) and parents not transporting the child to school or leisure activities (25, 26). Regarding contextual factors, previous research has determined that PA may be encouraged by access to facilities such as ballparks or playgrounds (27), traffic safety (28, 29), and the availability of gardens (30). The present study is the first to consider all these potential predictors of PA, examining objectively measured time spent in MVPA from age 6 to 10 in a population sample.

## Materials and methods

### Design and sample

The Trondheim Early Secure Study (TESS) comprises participants of two birth cohorts (born in 2003 or 2004) of children living in the city of Trondheim, Norway (~200,000 inhabitants) and their parents (*n* = 3,456). The participants were recruited when attending mandatory 4 year old health checkups at the municipal well-child clinics. The sample is comparable to the Norwegian population regarding parental level of education (31), family situation (i.e., marital status etc.) (32, 33) and children's BMI (34). 49.1 % of the recruited children where boys. Participants received a letter of invitation together with the Strength and Difficulties Questionnaire (SDQ) (35), a screening assessment for emotional and behavioral problems, which they brought to the health checkup. The SDQ measures emotional symptoms, conduct problems, hyperactivity/inattention, and peer relationship problems (20 items in total) and has shown excellent screening efficacy for children between 4 and 18 years old (36). The SDQ was used because the primary aim of the TESS study was to assess mental health. The recruitment process has been described in extensive detail elsewhere (37, 38), thus only a short description is provided here. Among those families who met the inclusion criteria (families with 4 year old children and parents with adequate proficiency in Norwegian) and were asked to participate (*n* = 3,016), 2,477 (82.1%) provided written informed consent in accordance with the Declaration of Helsinki. In order to increase sample variability, the SDQ total problem scores (20 items) were divided into 4 strata (cutoffs: 0–4, 5–8, 9–11, and 12–40), where drawing probabilities increased with increasing SDQ scores (0.37, 0.48. 0.70, and 0.89). A subsample of consenting families was drawn according to these procedures to participate in the TESS study (*n* = 1,250), and 997 (79.6%) participants were examined at the first wave of assessment. Thus, baseline data for the 2003 cohort was collected in 2007, whereas baseline data for the 2004 cohort was collected in 2008. Each data wave therefore takes 2 years to complete and all children are the same age when data is collected. At the reassessment when the child was 6 years old (T2; 1st grade), 795 families participated; 699 families participated in the 8-year assessment (T3, 3rd grade) whereas 702 families participated in the 10-year assessment (T4, 5th grade). PA measurements were included from T2 onwards; therefore, the data stem from children with valid PA data from T2 (*n* = 689), T3 (*n* = 607), and T4 (*n* = 684). A total of 800 children had usable PA data from at least one measurement point (i.e., T2-T4) and thus constitute the analytical sample. The parent not attending the data collection at the university completed a questionnaire about his/her own PA. The characteristics of the sample are shown in Table 1. Attrition analysis revealed that parents who participated at T3 spent slightly more time outside with their children at T2 compared with the non-participating parents at T3 (i.e., those who did participate at T2, but dropped out) [1.08. vs. 0.821 h per day, *t*_(1, 692)_ = 2.93, *P* = 0.02], and the participating children watched more TV at T2 compared with the children not participating at T3 [1.14 vs. 1.04 h per day, *t*_(1, 692)_ = 2.93, *P* = 0.02; but the total effect of these factors was minimal, Cox & Snell *R*^2^ = 0.014]. Attrition at T4 from T3 and T2 were unaffected by the child's level of PA and by the remaining study variables.

**Table 1:** Sample characteristics.

**Characteristics**	**%**
**GENDER OF CHILD**	
Male	49.8
Female	50.2
**GENDER OF INTERVIEWED PARENT**	
Male	18.9
Female	81.1
**ETHNIC ORIGIN OF BIOLOGICAL MOTHER**	
Norwegian	93.0
Western countries	6.8
Other countries	0.3
**ETHNIC ORIGIN OF BIOLOGICAL FATHER**	
Norwegian	93.0
Western countries	6.5
Other countries	0.5
**INTERVIEWED PARENT'S SOCIOECONOMIC STATUS**	
Leader	12.5
Professional, higher level	36.7
Professional, lower level	36.2
Formally skilled worker	14.1
Farmer/fisherman	0.0
Unskilled worker	0.6

The research procedures were approved by the Regional Committee for Medical and Health Research Ethics, Mid-Norway.

### Measurements

#### Moderate and vigorous physical activity

The children were instructed to wear an ActiGraph GT3X accelerometer (Manufacturing Technology Incorporated, Fort Walton Beach, FL, USA) for 7 consecutive days, 24 h a day, and only to take it off when bathing or showering. Only daytime activity was included. Following the recommendations, and common practice in child studies as summarized by Cain and colleagues, sequences of consecutive zero counts lasting ≥20 min were interpreted as non-wear time (39). Also following recommendations (40) and common practice in child studies (39) only participants with ≥ 3 days of recordings with ≥ 480 min of activity per day were included. Because young children's activity is often intermittent with short bursts, we applied 10 s epoch length, which is the length most commonly used in child studies (39). We further applied the Evenson et al. MVPA cut-off point of ≥2,296 counts per minute (41), as this cut-off is considered superior for children in middle childhood (42). Data were processed using accelerometer analysis software (ActiGraph LLC, Pensacola, FL, USA).

#### Child factors

The parents were asked whether their child participated in organized sports (No/Yes). They were also asked to estimate the total amount of time the child spent outside and in front of screens (“TV,” “Computers,” “Hand-held devices,” and “Gaming machines”) in minutes per day. The children reported on their athletic self-concept using the Children's Self-Description Questionnaire I, comprising of 8 items(α = 0.77), (e.g., “I am good at sports” using a five-point scale ranging from “true” to “wrong” (43). In addition to capture self-reported screen time, objectively measured sedentary behavior was measured by the accelerometer, where minutes per day with ≤ 100 counts per minute was considered sedentary activity, a cut-off that is widely used and has excellent classification accuracy (42).

#### Family factors

Each parent completed the International Physical Activity Questionnaire (IPAQ) which is a 31 item questionnaire widely used for monitoring physical activity in 18–65 year-olds (44). The particing parents were asked to estimate time engaged in various activities covering four domains of physical activity; work-related, transportation, housework/gardening and leisure-time activity (including exercise and sport participation). Data was summed within each category, and the number of minutes of MVPA per day was calculated according to standard procedures (44). Additionally, parents also reported (i) how many days they spent at least 10 min outside with the child; (ii) the total amount of time spent outside with the child on weekdays and weekends; (iii) and the means of transporting children to school, including walking, on each of the preceding 5 workdays. Active transportation was calculated as the mean number of days per week the child walked or bicycled to school.

#### Contextual factors

Socioeconomic status was measured by parental occupation, which was coded on a 6-point scale according to the International Classifications of Occupation (45): unskilled workers, farmers/fishermen, skilled workers, lower professionals, higher professionals and leaders. In two-parent household, the higher occupational status of the two parents were used. Parents reported whether their residence had a garden and were requested to estimate how long it would take them to reach a ballpark, playground, or other areas where the child could play, using an eight-point scale with responses ranging from “0 to 2 min” to “More than 2 h.” The parents were also asked to report how safe from traffic their child was when playing just outside the house, with options ranging from 1 (“Very unsafe”) to 4 (“Very safe”).

Data on all measures were collected biennially (age 6, 8, and 10).

### Statistical analyses

To adjust for all potential unmeasured time-invariant confounders a fixed effects regression analysis (14) was applied within a structural equation modeling (SEM) framework using Mplus 7.31 (46). In the SEM model MVPA at ages 10 and 8, respectively, were regressed on child, family and contextual predictors (measured at 8 and 6, respectively) which were treated as time-varying factors, except for gender, which was considered a time-invariant observed variable. A latent time-*invariant* factor was created loading on MVPA at all ages. All predictors were allowed to correlate with each other. They were also allowed to correlate with the latent time-invariant MVPA factor, thereby adjusting their effect on observed MVPA for what they would have in common with (unmeasured) time-invariant factors causing MVPA. Applying fixed (or random) effect regression within SEM allows for testing and comparing model fit between alternative and nested models. A random effects model is a statistically more powerful model than a fixed effects model, but it implies that the predictors are uncorrelated with the latent time-invariant factor, a presupposition that may not be true. A random effects model was compared to a fixed effect model by means of testing differences in χ^2^. Because differences in χ^2^ do not follow a χ^2^ distribution when a robust maximum likelihood estimator is used, Satorra–Bentler's scaled χ^2^ was applied (47), which is a functional equivalent to the Hausmann test (14). An ordinary random effects model does not have the fixed-effect advantage of eliminating the impact of all unmeasured time-invariant confounders. However, a hybrid random effects model does (15) and we therefore tested such a hybrid model, fixing insignificant correlations between predictors and the time-invariant factor to zero. Because the impact of predictors may be different at different ages, we compared a model where the regression coefficients were fixed to be equal at all ages with a model where they were freely estimated. Missing data were handled with a full information maximum likelihood procedure. Due to oversampling, the results were weighted back with a factor corresponding to the number of children in the population in a particular stratum divided by the number of participants in that stratum to arrive at corrected population estimates. We applied a robust Maximum Likelihood estimator, which is robust to moderate deviations from normality and provides corrected error terms needed because of the oversampling.

## Results

Table 1 displays sample characteristics of the study variables. Note that some of the contextual factors had modest variance; most parents indicated that it would take them and their child 0–2 or 3–5 min to reach an area conducive to PA (e.g., ballpark) and that their perception of traffic safety was very high.

### Predictors of MVPA

Descriptives of study variables along with means, standard deviations and bivariate associations are portrayed in Table 2. With regards to child factors, children's time spent outside, sport activities, athletic self-concept, being a boy and time spent sedentary were bivariately associated with MVPA. Significant bivariate family factors comprised of time parents spent outside with their child and parents not transporting their children to school. Socioeconomic status and having a garden were the only contextual factors that were bivariately associated with MVPA.

**Table 2:** Descriptives of study variables and bivariate associations between MVPA and their predictors.

**Predictors**	**M (SD)^a^**	**Bivariate correlation**
**CHILD FACTORS**		
Gender (0 = boy, 1 = girl)	0.50 (0.50)	−15.26^***^
Child's outdoor time, hours per day	2.57 (1.96)	1.62^***^
Number of sports activities	2.09 (0.90)	1.88^**^
Screen time, hours per day	1.76 (0.80)	−1.29
Athletic self-concept (1–5)	33.92 (5.50)	0.68^***^
Height (cm)	134.11 (7.02)	0.07
Fat (kg)	5.44 (2.87)	0.05
Sedentary time, hours per day	9.24 (7.98)	−0.15^***^
**FAMILY FACTORS**		
Mother's MVPA, min per day	33.62 (39.39)	0.02
Father's MVPA, min per day	52.95 (63.73)	0.00
Parents outdoors with child, hours per day	0.82 (0.88)	4.17^***^
Active transportation to school, days per week	3.66 (1.82)	0.96^**^
**CONTEXTUAL FACTORS**		
Socioeconomic status (1–6)	4.63 (0.96)	1.92^**^
Time to ballpark (1–8)	2.01 (1.17)	0.57
Time to other recreational area (1–8)	1.33 (0.75)	−0.95
Traffic safety (1–4)	3.26 (0.68)	1.22
Garden (0 = No, 1 = Yes)	1.07 (0.26)	7.80^***^

*MVPA, Moderate and Vigorous Physical Activity*.

a *Because the analyses revealed no differing effects between predictors and outcomes across time, M and SD and correlations are based on mean scores across the available time points*.

** *p < 01*.

*** *p < 001*.

Results of the model fitting procedure testing for multivariate fixed effects regression analyses are presented in Table 3. A fixed effects model (M2) fitted the data reasonably well, and a random effects model (M3) fitted the data significantly worse. Inspecting the results from M2 revealed that only physical self-concept was associated with the fixed effect, varying from *B* = 8.53, *P* = 0.05 at age 6 to *B* = 12.59, *P* = 0.001 at age 10. We therefore tested a hybrid, fixed and random effects model where the correlation between physical self-concept and the time-invariant factor was set free whereas all other correlations with the latent fixed effect were fixed to 0 (M4). Such a model fitted the data well, and better than the fixed effects model (M2). In models M2-M4 the effects of predictors were fixed to be identical at all ages, but freeing these predictors in model M5 did not improve the fit. In sum, for power, fit, and parsimonious reasons model M4 was preferred.

**Table 3:** Results of model fitting procedure.

	**X^2^**	**df**	***P*-value**	**ΔX^2^**	**df**	***P*-value**	**RMSEA (95% CI)**	**SRMR**	**CFI**	**TLI**
M1: Baseline model	939.16	144	<0.001							
M2: Fixed effect, effect of predictors similar at all ages (compared to M1)	131.34	80	<0.001	871.85	64	<0.001	0.028 (0.019, 0.037)	0.010	0.940	0.892
M3: Random effect, effect of predictors similar at all ages (compared to M2)	204.73	126	<0.001	73.44	46	0.006	0.028 (0.021, 0.035)	0.014	0.908	0.895
**M4: Hybrid effect, effect of predictors similar at all ages (compared to M2)**	**180.35**	**120**	<**0.001**	**50.01**	**40**	**0.133**	**0.025 (0.017, 0.032)**	**0.013**	**0.929**	**0.915**
M5: Hybrid effect, effect of predictors vary between ages (compared to M4)	137.94	86	<0.001	43.30	34	0.132	0.027 (0.019, 0.036)	0.013	0.939	0.898

*Preferred model in bold. Non-significant correlations with predictors and the fixed effect set to 0. ΔX^2^ is corrected according to Satorra's procedure. RMSEA, Root Mean Square Error of Approximation; SRMR, Standardized Root Mean Square Residual; CFI, Comparative Fit Index; TLI, Tucker-Lewis Index*.

The effects of child, family and contextual predictors on MVPA in M4 are presented in Table 4. Note that all factors are adjusted for each other and that the effects of time-invariant confounders were ruled out. Regarding child factors, being a boy predicted more time in MVPA, as did more time spent outdoors. Time spent in sedentary activity was the only factor forecasting *less* time in MVPA in children. None of the family factors included in the model predicted children's MVPA. With respect to other contextual factors, however, both parent perceived traffic safety and having a garden predicted higher MVPA.

**Table 4:** Multivariate associations between MVPA and their predictors.

**Predictors**	***B* (95% C.I)**	**β**	***P***
**CHILD FACTORS**			
Gender (%, boys)	13.11 (15.61, 10.61)	0.27	0.001
Child's outdoor time, hours per day	1.21 (0.61, 1.81)	0.08	0.001
Number of sports activities	0.98 (-0.15, 2.10)	0.02	0.09
Screen time, hours per day	-1.00 (-2.44, 0.44)	−0.04	0.17
Athletic self-concept (1–5)	0.23 (-0.03, 0.48)	0.05	0.08
Height (cm)	0.10 (-0.08, 0.28)	0.04	0.29
Fat (kg)	-0.22 (-0.68, 0.24)	−0.04	0.35
Sedentary time, hours per day	-0.15 (-0.18,-0.13)	−0.43	0.001
**FAMILY FACTORS**			
Mother's MVPA, min per day	0.00 (-0.03, 0.03)	0.00	0.93
Father's MVPA, min per day	0.00 (-0.02, 0.02)	0.01	0.77
Parents outdoors with child, hours per day^a^	0.98 (-0.54, 2.49)	0.04	0.21
Active transportation to school, days per week	0.24 (-0.34, 0.81)	0.02	0.42
**CONTEXTUAL FACTORS**			
Socioeconomic status (1–6)	1.00 (-0.07, 2.08)	0.04	0.07
Time to ballpark (1–8)	0.88 (-0.06, 1.83)	0.04	0.07
Time to other recreational area (1–8)	0.11 (-1.19, 1.41)	0.00	0.87
Traffic safety (1–4)	2.46 (0.88, 4.05)	0.07	0.002
Garden (0 = No, 1 = Yes)	6.76 (2.59, 10.97)	0.08	0.002

*Hybrid fixed and random effects model. MVPA, Moderate and Vigorous Physical Activity*.

a *Parents outside with child was only measured when children were 6 and 8 years of age, as parents spend less time outside with their offspring with increased age*.

## Discussion

To promote MVPA in childhood, multilevel factors affecting MVPA need to be identified. The present research examined child-, parent and contextual predictors of MVPA in a community sample of 6-year olds followed up biennially until the age of 10. This is the first study to investigate such relationships in children using objectively measured MVPA while applying a fixed effects method, which controls for all potential time-invarying factors (e.g., genetics, common method effects).

The results revealed that the amount of time the child spent outdoor strongly increased MVPA; this is not surprising given that young children's rate of MVPA is up to 10 times higher outdoors than indoors (48). However, we build upon and extend prior work (49) by showing that time spent outdoors predicts children's MVPA (50) upon accounting for a range of potentially confounding variables and all time-invariant factors. Increasing the time children spend outside would therefore appear to serve as a powerful intervention for increasing MVPA. Boys were more active compared to girls, which is in accordance with previous studies measuring MVPA with accelerometers (51). Sedentary behavior was the only factor that predicted less MVPA in children. Also considering that sedentary behavior have detrimental effects on a range of health-related outcomes in children, independent of their PA levels, interventions should aim to promote less sedentary behavior in children (52).

Offspring of parents who reported having a garden and perceived high levels of traffic safety displayed increased time on MVPA. Characteristics of the physical neighborhood environment (e.g., gardens, street environment, and playground facilities) have also previously been shown to be associated with more MVPA in children. One earlier study have tracked children's bouts of MVPA using Global Positioning System and found access to gardens to be of particular importance among the physical characteristics captured (30). Further, even though objective and subjective evaluations of traffic safety are more independent than one might expect, others have also found parent perceived traffic safety to be associated with higher levels of PA in children (10), with the reverse being true of low perceived traffic safety (29). Recall however, that we also found time spent outside to predict higher levels of MVPA in our multivariate approach, indicating that time spent outside and parental perceived traffic safety affect children's MVPA independent of each other. One potential explanation could be that when parents perceive their neighborhood as unsafe, they may constrain children's activity and hence limit children's opportunities for MVPA. However, they may still facilitate activity within more traffic safe areas, which may explain why time spent outside, and gardens predicted increased MVPA.

Even though previous research has found several family factors to be related to children's MVPA (5), none of the family factors considered in this inquiry proved to be significantly related to future MVPA. Further, although several child- and contextual factors earlier shown to be associated with children's MVPA were included (20, 24) (i.e., child's physical self-concept and parents not transporting their children to school or leisure activities), none of these were predictive of MVPA. This may be due to the fact that the method applied, in contrast to many of those employed in prior MVPA work (2, 8, 10), adjusts for the effect of several measured child, family and contextual factors while also adjusting for all time-invariant confounders. Consider in this regard that in accordance with other findings (24) parental time spent outside with the child was associated with child MVPA in the bivariate analyses, but when adjusting for child- and other family- and contextual factors, there were no significant effect of parental time spent outside on child MVPA. These findings underscore the need for multivariate analyses which also adjust for unmeasured confounders.

### Study limitations and strengths

This study had many strengths, including a large representative sample of children and objectively measured MVPA. Self-and proxy reports of MVPA correlate only modestly with objectively obtained values (53). It is therefore beneficial, if not critical, to employ objective measurements of PA, a methodological requirement very few population studies of children have fulfilled. Additionally, perhaps the most important strength of this study was the fixed effects regression approach that accounted for all time-invariant confounding factors. Like most other research, the work presented herein was not without limitations, and the results should be interpreted in the context of these. Even if the influence of time-invariant factors (e.g., trait-like reporting bias and seasonal changes, genetics) was ruled out, it is possible that uncontrolled confounding factors that are not time-invariant still could have affected the results. To illustrate: It is possible; even likely, that changes in peer relations may imply changes in outdoor time and changes in activities associated with MVPA (i.e., new friends being into playing soccer vs. playing videogames) are transient in childhood (54).

## Conclusion

Even though previous research has identified a range of predictors of MVPA in children, the lack of adjustment for confounders make the importance of these findings uncertain with respect to preventive efforts. Adjusting for measured time-varying and unmeasured time-invariant confounders we therefore extend former observational studies by narrowing the gap between mere prediction and causal inference and identify a restricted set of predictors of MVPA in children. Although in need of replication, our results indicate that promotion of MVPA in children should incorporate and encourage outdoor activities, and decrease sedentary behavior, especially in girls who are significantly less active compared to boys. Facilitating certain built environments in neighborhoods such as access to gardens and safe areas traffic wise may also promote more MVPA in children.

## Author contributions

TZ-T drafted the original manuscript, performed the data analysis, and approved the final manuscript as submitted. SS contributed to the statistical analyses, reviewed and revised the original manuscript, and approved the final manuscript as submitted. LW conceptualized and designed the study, contributed to the data analyses, reviewed and revised the original manuscript, and approved the final manuscript as submitted.

### Conflict of interest statement

The authors declare that the research was conducted in the absence of any commercial or financial relationships that could be construed as a potential conflict of interest.

## Abbreviations

PA: Physical activity
MVPA: Moderate to Vigorous Physical Activity
TESS: Trondheim Early Secure Study
SDQ: Strength and Difficulties Questionnaire
IPAQ: International Physical Activity Questionnaire.
